# Multidimensional factors of burnout in general practice: a cross sectional survey

**DOI:** 10.3399/BJGPO.2023.0171

**Published:** 2024-05-15

**Authors:** Marie Bayot, Anke Boone, Lode Godderis, Anne-Laure Lenoir

**Affiliations:** 1 Department of Clinical Sciences, Université de Liège, Liège, Belgium; 2 Centre for Environment and Health, Katholieke Universiteit Leuven, Leuven, Belgium; 3 Idewe, External Service for Prevention and Protection at Work, Heverlee, Belgium

**Keywords:** Belgium, emotional intelligence, general practitioners, job burnout, mental health, self-compassion, social support, working conditions

## Abstract

**Background:**

GPs are particularly vulnerable to job burnout. Tailored prevention and intervention strategies are needed.

**Aim:**

To investigate organisational, interpersonal, and individual factors contributing to exhaustion and disengagement at work among GPs.

**Design & setting:**

We conducted a cross-sectional study in a sample of Belgian GPs.

**Method:**

A total of 358 doctors (73% females, 301 with complete data) completed an online anonymous questionnaire assessing job burnout, psychosocial characteristics of the work environment, perceived social support in the private domain, emotional competence, and self-compassion.

**Results:**

GPs reported moderate levels of exhaustion and disengagement. Regression models showed that included factors jointly explained 69% of the variance in exhaustion and 63% in disengagement. Exhaustion was significantly predicted by female sex (*β* effect size = −0.1), high perceived emotional demands (*β* = 0.19), as well as low self-compassion (*β* = −0.14) and low emotional competence (*β* = 0.09). Disengagement was significantly predicted by low seniority (*β* = −0.12) and limited opportunities for development (*β* = −0.16). Both exhaustion and disengagement were predicted by low perceived quality of work (*β* = −0.19 and −0.14, respectively), meaning of work (*β* = −0.17 and −0.31, respectively), and role clarity (*β* = 0.09 and 0.12, respectively), as well as high perceived work–life conflict (*β* = 0.46 and 0.21, respectively). Moreover, GPs working in a multidisciplinary group reported lower levels of exhaustion and disengagement than those working in a monodisciplinary group or a solo practice, and this difference was associated with factors such as work–life conflict.

**Conclusion:**

Organisational, interpersonal, and intrapersonal factors interact to predict a substantial part of burnout in general practice. The most significant risk factors were perceived work–life conflict and poor meaning of work. Policymakers should work to support more sustainable practices based on the specific needs and constraints reported by GPs.

## How this fits in

Many factors influence individuals’ mental health at work. We need to understand the multidimensional determinants of burnout among GPs to tailor prevention and intervention. More particularly, to our knowledge there is no evidence on the influence of psychological factors and their potential interaction with work-related factors. This cross sectional survey found that perceived quality of work, meaning of work, role clarity, and work–life conflict were associated with GP burnout. Exhaustion was specifically associated with sex, perceived emotional demands, self-compassion, and emotional competence, whereas seniority and opportunities for development only had a significant effect on disengagement. Moreover, GPs working in a multidisciplinary group (as compared with a monodisciplinary group and solo practice) were the least vulnerable to burnout and reported the least work–life conflict. Future studies should investigate moderating variables, as well as causality, and extend this investigation to GP trainees. Policymakers should use these findings to support more sustainable practices.

## Introduction

Job burnout, which entails emotional exhaustion, disengagement, and a low level of personal accomplishment,^1^ has gained increasing attention in the scientific and political realms over the past 50 years. While rising burnout rates have challenged the work community as a whole, healthcare workers have stood out for their particular vulnerability to burnout, and the deleterious impact it has on their health,^2^ their quality of care,^3^ and health systems’ sustainability.^4,5^ Among doctors, trainees, junior physicians, and GPs have been identified as most prone to burnout.^6–9^ According to a recent international meta-analysis, burnout prevalence estimates for GPs vary between 6% and 33%.^10^

Burnout in healthcare workers has been linked to some of the features of working in health care, such as being exposed to human suffering and having on-call duties. Although studies have focused on these professional factors more, individual and psychological factors also have a significant impact on the risk of burnout and individuals’ sense of wellbeing.^11,12^ For example, perfectionism (the tendency to set very high performance standards for oneself) induces higher levels of daily stress.^13,14^ Conversely, self-compassion (the tendency to welcome one’s own experiences of failure with understanding and hindsight) greatly reduces the risk of burnout.^15,16^ Similarly, emotional intelligence acts as a buffer against the deleterious impact of negative emotions on burnout.^17^

Despite a growing body of literature, the factors involved in burnout specifically in GPs remain underexplored. Risk factors include sociodemographic characteristics (for example, sex and number of children), which are not modifiable but important to design tailored interventions; practice characteristics, such as emotional demands (for example, dealing with the psychosocial components of general medicine); high workload; time pressure during visits; chaotic working conditions (that is, disordered and/or tense); prolonged sitting; uncertainty; low work control; practice-related economic constraints; social characteristics, such as practice culture (for example, isolation, lack of support, poor quality of relationships); patients’ expectations; exposure to workplace violence; working in a rural area; and working in a group practice (as compared with solo practice).

Personal characteristics can also contribute to burnout, such as using few stress-regulating measures (for example, playing sports, talking to friends), insufficient physical activity, and difficulties in balancing one’s professional and private lives.^18–23^ Protective factors include benefitting from group supervision and teamwork quality.^24,25^ To our knowledge, however, there is no evidence regarding the influence of psychological factors and their potential interaction with work-related factors. Examining individual and work-related factors together would enable comparisons to be made in their respective associations with burnout. Therefore, the aim of this study was to jointly investigate organisational, interpersonal, and individual burnout risk and protective factors among GPs, to support tailored prevention and intervention.

## Method

### Participants

From the convenience sample of 358 Belgian doctors who participated in the online study, 301 completed the whole questionnaire, which is sufficient to test our regression model according to standard rules of thumb (that is, 30 observations for one independent and one dependent variable, plus an additional 10 observations for every additional independent variable included: 30 + (25*10) = 280). Seventy-two per cent of participants were female, and mean seniority was 16.76 years (standard deviation [SD] = 12.14). Both main language communities (Dutch-speaking and French-speaking) were represented (49% and 51%, respectively). Among participants, 45% reported working in a solo practice, 26% in a monodisciplinary group, and 29% in a multidisciplinary group. Practice location varied from rural to urban (12% reported working in communities of <10 000 people, 33% in communities between 10 000–20 000, 19% in communities between 20 000–30 000, and 36% in communities >40 000).

### Procedure

Participants were recruited via email and newsletters from academic and professional institutions. The invitation to participate included a short description of the study and a link to the survey, hosted on Qualtrics, a secure online data collection software. After the request for informed consent, sociodemographic and study variables were measured with a forced-choice format to prevent missing data during completion.

### Measures

We used the Oldenburg Burnout Inventory (OLBI),^26^ in its original Dutch version and its French version,^27^ to measure self-reported exhaustion and disengagement at work. Psychosocial characteristics of the work environment were assessed using 38 items that applied to the context of general practice, selected from the third version of the Copenhagen Psychosocial Questionnaire (COPSOQ-III).^28^ Perceived social support in the private domain was measured using the Multidimensional Scale of Perceived Social Support (MSPSS), in its Dutch^29^ and French^30^ versions. Emotional competence was measured using the Short Profile of Emotional Competence (S-PEC).^31^ Self-compassion was measured using the Self-Compassion Scale – Short Form (SCS-SF), in its original Dutch version^32^ and in its French version.^33^ A detailed presentation of measures can be found in the Supplementary Files.

### Data analysis

As risk and protective factors may influence exhaustion and disengagement differently, we ran analyses for both these dimensions of burnout separately. To explore unconditional effects of each predictive factor on exhaustion and disengagement, we performed Pearson’s bivariate correlations (see Supplementary Table S1). To test the added value of other variables, above and beyond sociodemographic factors, and investigate their respective weight in the prediction of job burnout dimensions, we used a hierarchical linear regression model. In step 1, we entered sex, seniority, language community, type of practice, and workplace population as sociodemographic control factors (Model 1). In step 2, we added psychosocial characteristics of the work environment (COPSOQ-III), emotional competence (S-PEC), perceived social support in the private domain (MSPSS), and self-compassion (SCS-SF) as predictive factors (Model 2). Post hoc analyses were performed for categorical variables with a significant *β*. As type of practice was significantly associated for both exhaustion and disengagement in Model 1, we conducted one-way analysis of variance (ANOVA) tests and post hoc comparisons (Fisher's least significant difference) to compare the three categories (that is, solo practice, monodisciplinary group, and multidisciplinary group) on all variables. All analyses were conducted using IBM SPSS Statistics (version 25.0).

## Results

GPs (*n* = 358) reported moderate levels of exhaustion (mean [*M*] exhaustion score = 2.68, SD = 0.55) and disengagement (*M* = 2.38, SD = 0.5).

### Regression analyses

As shown in Table 1, the hierarchical linear regression models showed a significant contribution of sociodemographic factors to the prediction of exhaustion and disengagement (Model 1). The model supplemented with other potential predictors (Model 2) accounted for much more variance than Model 1.

**Table 1.** Summaries of regression models for exhaustion and disengagement among GPs (*N* = 283)

	Exhaustion					Disengagement				
Model	R^2^	AdjR^2^	ΔR^2^	F (df)	*P* value	R^2^	AdjR^2^	ΔR^2^	*F* (df)	*P* value
1	0.05	0.03		2.6 (5, 277)	0.03	0.11	0.09		6.77 (5, 277)	<0.001
2	0.69	0.66	0.65	25.91 (21, 256)	<0.001	0.63	0.59	0.52	17.12 (21, 256)	<0.001

AdjR^2^ = adjusted R^2^. df = degrees of freedom.

Variables that remained significantly associated with exhaustion in the final model were language community (*β* = −0.11, *P* = 0.01, 95% confidence interval [CI] = −0.23 to −0.03), sex (*β* = −0.10, *P* = 0.01, 95% CI = −0.23 to −0.03), emotional competence (*β* = 0.09, *P* = 0.03, 95% CI = 0.01 to 0.22), self-compassion (*β* = −0.14, *P* = 0.003, 95% CI = −0.18 to −0.04), perceived quality of work (*β* = −0.19, *P*<0.001, 95% CI = −0.01 to −0.003), emotional demands (*β* = 0.19, *P*<0.001, 95% CI = 0.004 to 0.01), meaning of work (*β* = −0.17, *P* = 0.001, 95% CI = −0.1 to −0.002), role clarity (*β* = 0.09, *P* = 0.04, 95% CI = 0.001 to 0.01), and work–life conflict (*β* = 0.46, *P*<0.001, 95% CI = 0.01 to 0.01). See Supplementary Table S2 for a detailed presentation of all regression coefficients.

Variables that remained significantly predictive of disengagement in the final model were seniority (*β* = −0.12, *P* = 0.02, 95% CI = −0.01 to −0.001), perceived quality of work (*β* = −0.14, *P* = 0.004, 95% CI = −0.01 to −0.001), opportunities for development (*β* = −0.16, *P* = 0.012, 95% CI = −0.01 to −0.001), meaning of work (*β* = −0.31, *P*<0.001, 95% CI = −0.1 to −0.01), role clarity (*β* = 0.12, *P* = 0.01, 95% CI = 0.001 to 0.01), and work–life conflict (*β* = 0.21, *P*<0.001, 95% CI = 0.002 to 0.01). See Supplementary Table S3 for a detailed presentation of all regression coefficients.

### Post hoc analyses

One-way ANOVAs outlined a significant effect of sex for exhaustion (*F*(1, 356) = 4.82, *P* = 0.03), with women (*M* = 2.72, SD = 0.54) reporting significantly higher levels than men (*M* = 2.58, SD = 0.57), as well as a significant effect of type of practice on both exhaustion (*F*(2, 357) = 3.34, *P* = 0.04) and disengagement (*F*(2, 357) = 8.19, *P*<0.001). Multiple comparisons showed that GPs working in a multidisciplinary group reported significantly lower levels of exhaustion and disengagement than GPs working in a monodisciplinary group (*P* = 0.018, 95% CI = −0.34 to −0.03; *P* = 0.009, 95% CI = −0.32 to −0.05, respectively) or a solo practice (*P* = 0.035, 95% CI = −0.28 to −0.01; *P*<0.001, 95% CI = −0.36 to −0.12, respectively).

**Figure 1.**
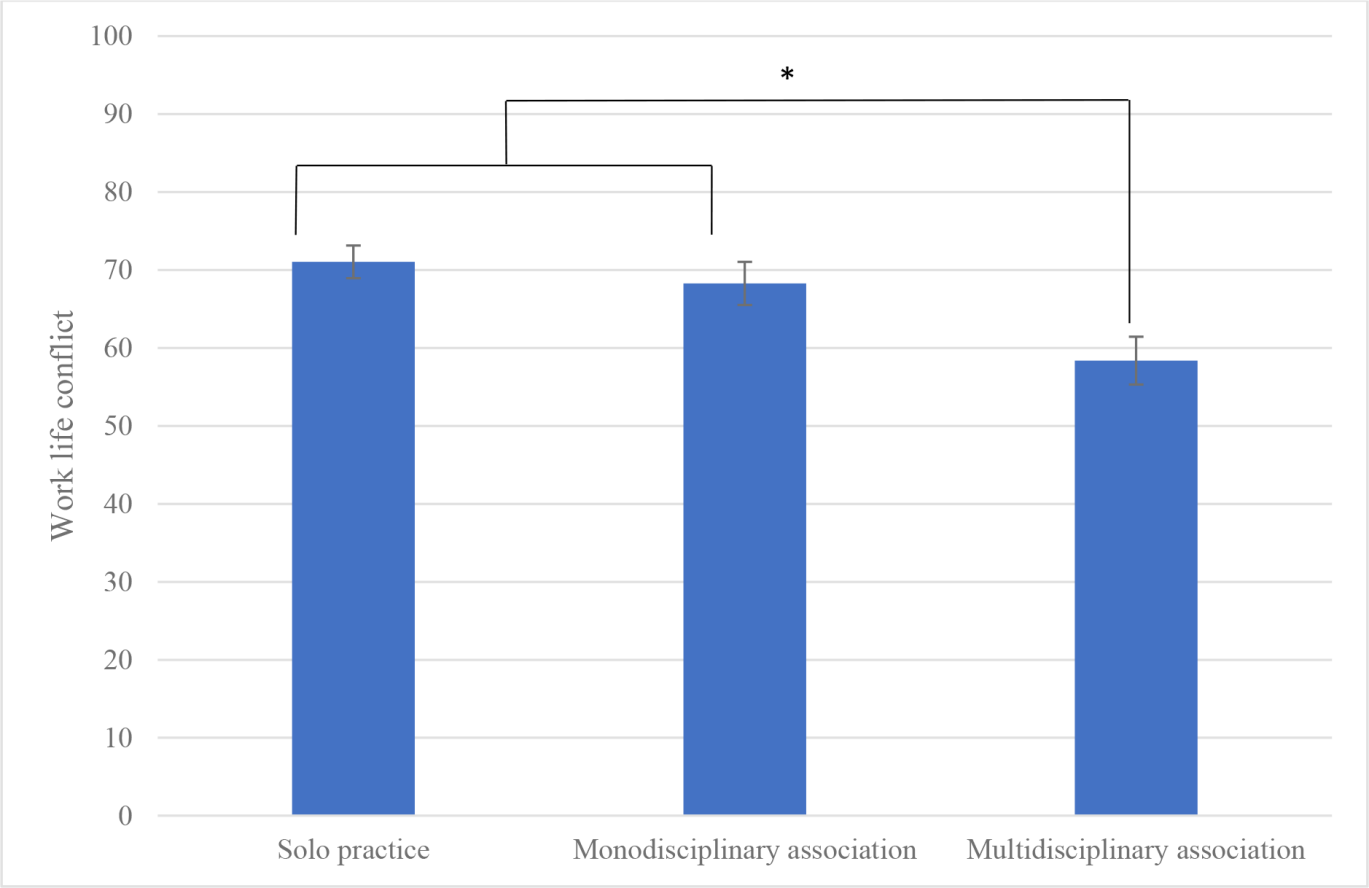
Reported work–life conflict (COPSOQ-III) by type of practice among GPs. Error bars represent standard error. Asterisk indicates significant difference.

One-way ANOVAs with predictive factors further showed significant effects of type of practice (see Supplementary Table S4 for descriptive data and test results). In comparison with GPs working in a multidisciplinary group, GPs working in a solo practice reported more illegitimate tasks (*P* = 0.006, 95% CI = 2.88 to 16.6), and less social support from family (*P* = 0.011, 95% CI = −0.85 to −0.11), friends (*P*<0.001, 95% CI = −1.16 to −0.41), or a significant other (*P* = 0.008, 95% CI = −0.9 to −0.13). They also reported less social support from colleagues (*P*<0.001, 95% CI = −26.78 to −14.58), a poorer sense of community at work (*P*<0.001, 95% CI = −26.44 to −14.66), fewer opportunities for development (*P* = 0.002, 95% CI = −13.4 to −2.99), less meaning of work (*P* = 0.012, 95% CI = −11.46 to −1.41), and less recognition (*P*<0.001, 95% CI = −15.26 to −4.92). Working in a monodisciplinary group rather than a solo practice was associated with the following protective factors: higher social support from friends (*P* = 0.032, 95% CI = −0.82 to −0.04), control over working time (*P* = 0.005, 95% CI = −11.95 to −2.18), social support from colleagues (*P*<0.001, 95% CI = −22.56 to −10.12), sense of community at work (*P*<0.001, 95% CI = −25.32 to −13.32), and recognition (*P* = 0.002, 95% CI = −13.61 to −3.07). The protective factors associated with working in a multidisciplinary group rather than a solo practice or a monodisciplinary group were: lower demands for hiding emotions (*P* = 0.001, 95% CI = −13.04 to −3.43; *P* = 0.006, 95% CI = −13.05 to −2.22, respectively) and lower work–life conflict (*P*<0.001, 95% CI = −19.64 to −5.69; *P* = 0.014, 95% CI = −17.76 to −2.04, respectively) (see Figure 1).

## Discussion

### Summary

GPs exhibited moderate levels of exhaustion and disengagement based on cut-off scores from the literature.^34^ Data analysis revealed several risk and protective factors, with a small-to-medium effect size on job burnout. Risk factors for exhaustion were female sex, high perceived emotional demands, low self-compassion, and low emotional competence. Risk factors for disengagement were low seniority and limited opportunities for development. Both exhaustion and disengagement were predicted by low perceived quality of work, low meaning of work and role clarity, high perceived work–life conflict, and practice type. Specifically, working in a multidisciplinary group (as compared with a monodisciplinary group and solo practice) was a protective factor against job burnout. Interestingly, working in a multidisciplinary group influenced several factors associated with exhaustion and disengagement at work, some of which are inherent to working in a group (for example, social support from colleagues, sense of community at work in a group practice), while others were unexpected (for example, demands for hiding emotions) and call for further investigation with, for example, qualitative methods.

### Strengths and limitations

Our regression models explained a substantial portion of variance in exhaustion (69%) and disengagement (63%) among GPs, leading us closer to our ultimate objective of identifying modifiable factors (in contrast with demographic factors) for burnout prevention and intervention in general practice.

However, the main limitation of this study lies in its cross-sectional nature, which precludes us from making any claims of causality. Regarding the effect of type of practice, several criteria that may have nuanced our results (for example, remuneration mode, private versus public sector) were not measured. Moreover, the self-reported nature of our data precludes us from excluding social desirability bias in our results. Finally, the timing of our data collection, which occurred shortly after the COVID-19 crisis, which had a measurable impact on GPs’ wellbeing and practice,^35^ may have influenced our findings.

### Comparison with existing literature

Our findings confirm those from previous studies of GP burnout; that is, the effects of emotional demands, sex (women being more at risk than men), and work–life balance.^18–20^ They also extend findings from studies of burnout in other healthcare workers to GPs; that is, the protective effects of perceived meaning of work,^36^ emotional competence,^17^ and self-compassion.^37^ Our study supplements previous findings by highlighting the effects of perceived opportunities for development, quality of work, and role clarity.

In contrast, some of our findings contradict previous observations. For example, type of practice (group versus solo) was not significantly related to burnout in a study among Swiss primary care doctors,^19^ and was inversely associated with burnout (that is, doctors working in group practices were more vulnerable to burnout) in a study of German GPs.^22^ While these discrepancies may be attributed to actual differences between samples (for example, inherent to political and organisational variations from one country to another), we hypothesise that this may derive from different operationalisations of the variable (that is, the criteria of categorisation). Indeed, the effect of type of practice we observed may have been masked if specific categories (for example, monodisciplinary and multidisciplinary groups) had been merged, as in the above-mentioned studies. Beyond type of practice, the quality of teamwork plays a key role in mitigating burnout in group practices.^25^ Measuring this variable, as was done in our study (for example, perceived sense of community at work, social support from colleagues and recognition), may therefore bring greater clarity to the mixed results observed.

Finally, our findings point to specific factors associated with exhaustion on the one hand, and disengagement on the other, in GPs. Interestingly, exhaustion was specifically associated with factors that either deplete (perceived emotional demands) or replenish (self-compassion and emotional competence) emotional resources, further showing the emotional mechanism behind this burnout dimension.^1^ Disengagement was specifically associated with opportunities for development, which aligns with the literature on motivation and engagement at work.^38^

### Implications for research and practice

At the research level, moderating variables at play in the effect of burnout risk factors, as a function of personal or work characteristics, should be investigated, to fully understand the mechanisms at stake and better prevent burnout in GPs. Furthermore, since GP trainees are exposed to specific working conditions, inherent to the educational environment (for example, less autonomy, lower levels of responsibility, potential role of the relationship with supervisors),^39^ our findings cannot be generalised to that particular population. Future studies are needed to identify burnout risk and protective factors among GP trainees to inform the design of tailored prevention and intervention. Studying GP trainees would also enable the examination of the role of generational and cultural factors in burnout; for example, through an effect on work–life conflict.

At the practice level, policymakers should acknowledge organisational (for example, work–life conflict), interpersonal (for example, quality of teamwork), and intrapersonal (for example, emotional competence) burnout factors to support a more sustainable healthcare system. Concomitantly, evidence-based programmes tailored to GPs’ specific needs and constraints should be embedded into the continuing education curriculum. For example, interventions promoting physical and mental health (such as mindfulness-based programmes) could be implemented.^40,41^

## Data

The dataset relied on in this article is available from the corresponding author on reasonable request.
